# Epidemiology and Characteristics of SARS-CoV-2 Variants of Concern: The Impacts of the Spike Mutations

**DOI:** 10.3390/microorganisms11010030

**Published:** 2022-12-22

**Authors:** Théophile Cocherie, Karen Zafilaza, Valentin Leducq, Stéphane Marot, Vincent Calvez, Anne-Geneviève Marcelin, Eve Todesco

**Affiliations:** Institut Pierre Louis d’Épidémiologie et de Santé Publique (iPLESP), INSERM, Sorbonne Université, Assistance Publique-Hôpitaux de Paris (AP-HP), Hôpital Pitié-Salpêtrière, Service de Virologie, 75013 Paris, France

**Keywords:** SARS-CoV-2, COVID-19, Spike glycoprotein, SARS-CoV-2, immune evasion, transmissibility, SARS-CoV-2 variants, variants of concern

## Abstract

SARS-CoV-2 expresses on its surface the Spike protein responsible for binding with the ACE2 receptor and which carries the majority of immunodominant epitopes. Mutations mainly affect this protein and can modify characteristics of the virus, giving each variant a unique profile concerning its transmissibility, virulence, and immune escape. The first lineage selected is the B.1 lineage characterized by the D614G substitution and from which all SARS-CoV-2 variants of concern have emerged. The first three variants of concern *Alpha*, *Beta*, and *Gamma* spread in early 2021: all shared the N501Y substitution. These variants were replaced by the *Delta* variant in summer 2021, carrying unique mutations like the L452R substitution and associated with higher virulence. It was in turn quickly replaced by the *Omicron* variant at the end of 2021, which has predominated since then, characterized by its large number of mutations. The successive appearance of variants of concern showed a dynamic evolution of SARS-CoV-2 through the selection and accumulation of mutations. This has not only allowed progressive improvement of the transmissibility of SARS-CoV-2, but has also participated in a better immune escape of the virus. This review brings together acquired knowledge about SARS-CoV-2 variants of concern and the impacts of the Spike mutations.

## 1. Introduction

SARS-CoV-2 belongs to the *Coronaviridae* family, *Coronavirinae* subfamily, *Betacoronavirus* genus, and *Sarbecovirus* subgenus like SARS-CoV and other animal coronaviruses.

The name *Coronavirus* comes from the appearance of the viral particles under electron microscopy: it has a spherical structure and carries on its surface projections made of the Spike glycoprotein (S protein) which form a kind of crown.

It is an enveloped virus with a single-stranded, linear, non-segmented RNA with a positive polarity and a virus particle size of 100 nm. Human-to-human transmission of the virus occurs through the respiratory route, mainly by droplets emitted during breathing or coughing and by aerosols. The tropism of SARS-CoV-2 is mainly but not exclusively respiratory. The most common symptoms are fever, asthenia, and a dry cough. The presence of dyspnea, confusion, loss of appetite, and chest tightness may indicate severe SARS-CoV-2 infection.

### 1.1. Genome Organization and Proteins of SARS-CoV-2

The viral genome is approximately 30,000 base pairs long and consists of 10 open reading frames (ORFs). ORF1ab makes up two-thirds of the genome and encodes 16 non-structural proteins (NSP1 to NSP16). Four ORFs code for structural proteins: Spike (S), Envelope €, Membrane (M), and Nucleocapsid (N) proteins. The rest of the ORFs code for accessory proteins. The majority of NSPs make up the RNA-dependent replication polymerase complex (RdRp) and NSP14 contains a 3′-5′exoribonuclease with a proofreading action to correct errors made during viral replication [1]. The Spike protein is a glycoprotein organized as a homotrimer anchored in the viral membrane and is 1273 amino acids long [2]. It is composed of two functional subunits S1 and S2 separated by a furin cleavage site (FCS) at position 682–685 [3,4]. Cleavage at the FCS is due to a cellular furin within an infected cell during maturation of the neo-synthesized S proteins in the Golgi apparatus. It participates in reducing the stability of the S protein by removing the covalent bond between S1 and S2 and facilitates the conformational adaptation required for RBD presentation and binding to its receptor (the angiotensin-converting enzyme 2 ACE2) [5]. This cleavage may also be responsible for the early dissociation of S1 and S2 associated with a conformational change of S2, rendering the S protein nonfunctional and unable to bind to its receptor, blocking viral penetration [6,7]. The presence and conservation of the FCS in SARS-CoV-2 despite the loss of stability generated shows the importance of this site for viral fitness, highlighted also by the fact that the first mutation to spread and replace the ancestral strain, the D614G mutation, is the one whose effects mainly counteract this disadvantage [8]. The S1 subunit is responsible for binding to the ACE2 receptor while the S2 subunit is responsible for fusion of the viral and cell membranes following this binding [9]. S1 is composed of the receptor binding domain (RDB) flanked by an N-terminal domain (NTD) and a C-terminal domain (CTD) [10]. It has a high genetic diversity among coronaviruses [6]. The NTD of S1 carries a large number of immunodominant epitopes on its surface [11]. The RBD adopts two conformations within the S protein: a “down” position where the receptor binding motif (RBM) is packed between the S2 central helix, the S1-CTD of the same protomer and the S1-NTD of an adjacent protomer and an “up” position where the RBM is exposed with a rearrangement of the involved S1-CTDs and NTDs allowing stabilization of the S protein [10]. In the “down" position, the RBM is masked from its ACE2 receptor while in the “up” position it is exposed to its ACE2 receptor but also to the immune system. There is a natural balance between the “all-down” and “one-up” forms of protein S. The S2 subunit carries important domains for viral penetration such as a second furin cleavage site (S2’), the fusion peptide (FP), and the fusion peptide proximal region (FPPR), as well as the heptad repeat 1 and 2 (HR1 and HR2) separated by a central helix (CH), the transmembrane domain (TM), and the cytoplasmic domain (CT) [10]. S2 has two very different conformations: pre-fusion and post-fusion. S2 is maintained in the pre-fusion conformation by its interaction with S1. The post-fusion conformation is adopted after the two cleavages at the FCS and S2’ and the dissociation of S1 from S2 [7]. This conformational change is the most important mechanism of cell membrane fusion.

The E protein plays a role in the assembly phase of the viral particle [12]. The M protein binds to other structural proteins, allowing the assembly of the viral particle and participating in its virulence [12]. The N protein has a tubular structure with helical symmetry. It plays a role in the envelopment and protection of the viral RNA, the release of the viral particle, and the formation of the ribonucleic core [12].

### 1.2. The Viral Cycle of SARS-CoV-2

The ACE2 receptor is found on the extracellular face of plasma membranes of cells in the lung, arteries, heart, kidney, and digestive tract. After RBD–ACE2 binding, there are two routes of virus entry into the cell: either by direct fusion of the plasma and viral membranes or after uptake by endocytosis.

Direct fusion of the viral and cellular membranes requires the action of a cellular protease: the transmembrane serine protease 2 (TMPRSS2) cleaves the S protein at S2’, leading to the conformational change of the S homotrimer with dissociation of S1 and passage of S2 to its post-fusion conformation, where the FP is propelled and inserts into the cellular membrane. This insertion is followed by a folding of HR1 and HR2 which leads to the bringing together of FP and TM and thus of the viral and cellular membranes. It allows the creation of a membrane pore and passage of the viral particle contents into the cytoplasm of the newly infected cell [13,14,15].

In the case where the virus particle is endocytosed, the conformational change of S2 is initiated by the action of cellular cathepsins, such as cathepsin L, within the endolysosome and resulting in the release of viral RNA into the cell cytoplasm [14].

Replication of SARS-CoV-2 within the infected cell involves direct translation of single-stranded, positive-polarity RNA to synthesize a pp1ab polyprotein that has self-cleaving activity responsible for its proteolytic cleavage and the production of the 16 mature nonstructural proteins that will form the RdRp.

The replication of the genome is done through a single-stranded RNA of negative polarity and the synthesis of viral proteins is done by discontinuous transcription [16,17].

The neosynthesized viral proteins mature through the endoplasmic reticulum and then through the Golgi apparatus from which vesicles sprout. The replicated RNA binds to the N protein to form the nucleocapsid which is integrated into these vesicles through the interaction between the M and N proteins and then the viral particles are excreted into the extracellular medium.

## 2. Genetic Variability of SARS-CoV-2

Viruses are characterized by their constant genetic evolution to adapt their fitness to their host. The occurrence of mutations is random and linked to replication errors, editing mechanisms mediated by the host cell or genetic recombination in case of infection of the same host cell by several different viral strains [18]. These mutations can accumulate over time and it is the balance of their beneficial and deleterious effects that will guide the natural selection of the viral strain towards diffusion or extinction [19]. Viral pandemics are known to involve several levels of mutations leading to the selection of specific variants and the circulation of multiple viral strains, especially in the case of RNA viruses due to the lack of proofreading action by their RdRp.

The SARS-CoV-2 has the particularity, like other coronaviruses, of having an exonuclease activity, leading to a slower mutation rate than other RNA viruses. Nevertheless, it has shown a diversification of its genome characterized by the progressive accumulation of new mutations, due to its high circulation and the different selection pressures. These mutations are generally associated with an increase in the affinity of the RBD for its membrane receptor, a high replication rate, an increased infectivity and contagiousness, a more or less important virulence, or the appearance of an immune escape. The majority of the selected mutations are located in the gene coding for the S protein, due to its exposure to the immune system on the surface of the virus particle. The majority of immunodominant epitopes are carried by the NTD of the S1 of the S protein and resistance mutations alter the conformation of the S protein. These conformational changes may deleteriously impact other viral functions and other compensatory mutations may be associated with them to restore viral fitness. Indeed, some mutations that did not persist because of the deficits provided but reappeared associated with other mutations compensating for these deficits have been observed.

### Classification and Nomenclature of the Different Variants

The variants were classified by the World Health Organization (WHO) into several levels: under surveillance, of interest or of concern according to clinical, epidemiological, prevention and therapeutic criteria, and the level of evidence of their impact [20]. Some variants have also been reclassified according to their epidemiological evolution.

The nomenclature of the different SARS-CoV-2 variants has evolved in accordance with their identification method and the context in which they are mentioned.

The variants of concern (VOC), on which we focus on in this review are SARS-CoV-2 variants that have been demonstrated through a comparative assessment to be associated with one or more of the following changes at a degree of global public health significance: Increase in transmissibility or detrimental change in COVID-19 epidemiology OR increase in virulence or change in clinical disease presentation OR decrease in effectiveness of public health and social measures or available diagnostics, vaccines, therapeutics [20]. There are also the *Omicron* subvariants under monitoring that, according to phylogenetic analysis, belong to a currently circulating VOC AND shows signals of transmission advantage compared to other circulating VOC lineages AND have additional amino acid changes that are known or suspected to confer the observed change in epidemiology and fitness advantage as compared to other circulating variants [20].

Since May 2021, WHO decided to change the name of the existing variants to letters of the Greek alphabet [20]. The main idea behind these new names was to facilitate the public debate by having names that are easy to pronounce and remember but also to avoid the general public and the media using stigmatizing and discriminatory names. The scientific names continue to exist and are still used by the scientific community because they provide more information than their simplified version.

The PANGO nomenclature mainly uses the sequences available in the GISAID database and seeks to identify different lineages taking into account both evolution and epidemiological impact. Each identified lineage is designated by a prefix composed of a letter and followed by several suffixes separated by dots and composed of numbers.

The Nexstrain nomenclature identifies the different clades by phylogenetic and phylodynamic analysis [21]. Each major clade is named by a prefix indicating its year of emergence followed by a letter indicating the order in which each clade has been identified.

## 3. Circulation of SARS-CoV-2 Variants

SARS-CoV-2 is responsible for COVID-19 and the pandemic that has been ongoing since December 2019 which has caused more than 641 million cases and 6.6 million deaths worldwide as of 28 November 2022 [22].

Initially, two strains were described in China defining their own lineage: A and B [23]. Lineage B is the one that has spread the most around the world and is the source of the pandemic and of the main variants that appeared later.

The first cases of SARS-CoV-2 infection were suspected as early as 8 December 2019, in China in the Wuhan area. The discovery of SARS-CoV-2, published on 9 January 2020 [24], gave a name to the associated pathology: COVID-19. Despite the quarantine measures put in place, other cases were diagnosed outside of China, first in Thailand on 13 January 2020, and then on several continents, such as in the United States on 21 January 2020, in travelers returning from this region. In Europe, SARS-CoV-2 was initially found in France on 24 January 2020 and then in Italy on 30 January 2020.

The first cases diagnosed in Europe and America were infected by one of the two strains that circulated in Wuhan, specifically the B strain. However, as early as January 2020, the presence of the D614G mutation was observed in certain circulating strains, mainly in Europe. This mutation spread rapidly throughout the world, replacing the initial circulating strains. The associated variant was called B.1 (PANGO nomenclature) or 20A (Nexstrain nomenclature) [23]. Europe then became the new epicenter of the pandemic, joined later by North America. The first wave of SARS-CoV-2 infection affected all continents of the world, but to varying degrees and with national/regional disparities. The following waves of the pandemic affected all regions of the world, sometimes concomitantly or consecutively.

The second wave of SARS-CoV-2 infection, still linked to the B.1 lineage, took place between September and November 2020 in a context of lifting of isolation restrictions and gradual resumption of social and economic activities in the different countries.

At the end of 2020, the first three VOC, all from the B.1 lineage, were described, had spread, and were replaced the original strain [20,25,26]. They were characterized by the accumulation of mutations in the protein S gene. The first two VOC to be described were the *Alpha* variant (PANGO nomenclature: B.1.1.7), first detected in the UK in September 2020, and the *Beta* variant (B.1.351), first detected in South Africa in May 2020. These two variants were defined as VOC by the WHO on 18 December 2020 [20]. A third VOC, the *Gamma* variant (P.1), was described for the first time in Brazil in November 2020 and defined as a VOC by the WHO on 11 January 2021 [20]. The *Alpha* variant was mainly found in Europe and North America but also circulated on all continents. It circulated predominantly worldwide until May 2021. The *Beta* variant was mainly described in Europe and in the southern part of the African continent but was also found on all continents. The *Gamma* variant circulated mainly in North and South America and was not found in Africa [27]. These three variants were responsible for the third wave of COVID-19 worldwide between March and June 2021.

In March 2021, the *Delta* variant (B.1.617.2) was described in India and was defined as a VOC by the WHO on May 11, 2021 [20]. This variant had spread rapidly and had replaced the previous circulating VOC within a few months, to become the only variant circulating in an ultra-majority way in the world from September 2021. It was characterized by the more severe forms it caused, particularly among unvaccinated populations, as well as by the fact that it affected the young population, most of whom had previously been spared from severe forms of infection. The Delta variant was responsible for the fourth wave of COVID-19 in the world between July and October 2021.

The *Omicron* variant initially called B.1.1.529 and reclassified as BA.1 was first described on 2 September 2021 in South Africa. It had been the cause of a sudden emergence of positive cases since November 2021, in a region where the Delta variant was circulating weakly. It was rapidly defined as a VOC by the WHO on 26 November 2021 [20]. The *Omicron* variant BA.1 spread worldwide and replaced the *Delta* variant in a rapid manner, in only a few weeks during December 2021. Indeed, the *Delta* variant was still predominant on 13 December 2021, associated with 89% of the COVID-19 cases, but already represented only 50% of the cases on 20 December 2021 [28].

The *Omicron* variant BA.1 was responsible for the fifth wave of COVID-19 worldwide. The *Omicron* variant distinguishes itself by the appearance of sublineages, initially BA.2 which gradually replaced the *Omicron* variant BA.1. The BA.2 sublineage became predominant in March 2022 and then an ultra-majority in April 2022, before the appearance of the BA.4 and then the BA.5 sublineages in May 2022. The latter progressively replaced BA.2 and is responsible for the last two waves of infections.

Since July 2022, the BA.5 sub-variant has remained predominant but has evolved. Indeed, it is now divided into different sublineages, such as BF.7 or BQ.1* whose circulation is increasing. According to ECDC modelling, the circulation of BQ.1* in the European Union could reach 80% by the end of 2022 [29].

## 4. The Main Variants of SARS-CoV-2: Spike Mutations and Their Impacts

This section describes the clade B.1 and the VOC of SARS-CoV-2, their main Spike mutations, and the conformational, epidemiological, and clinical impact of the Spike mutations (cf. Table 1).

### 4.1. Clade B.1: D614G

The lineage named B.1 according to the PANGO nomenclature was characterized by a single mutation in the Spike gene: D614G [30]. This D614G substitution was first reported in January 2020 and spread rapidly in an immune-naive population.

Position 614 is located on the surface of the S1 subunit of the S protein and causes the disruption of a hydrogen bond within the homotrimer with a threonine at position 859 on S2, continuously opening a RBD in the “up” position [31]. This conformation increases its flexibility and promotes its binding to the ACE2 receptor [32,33,34]. Homotrimers in the “2-up” or even “3-up” conformation were also observed, further increasing the binding potential to the ACE2 receptor, even doubling the strength of the RBD–ACE2 binding in some studies [35].

The D614G substitution is also associated with greater thermodynamic stability of the homotrimer by intervening on the 630 loop, stabilizing interactions between the S1 and S2 subunits and reducing early dissociation of S1 and loss of S protein functionality [36,37].

The transmissibility of the D614G mutated strain was thus increased and contributed to its dissemination [32]. Nevertheless, other changes were reported: viral loads were higher related to a better viral replication in the upper airways which was associated with an increase in the prevalence of certain clinical signs (such as anosmia), and to a greater production of viral particles and thus a putative greater contagiousness [37]. However, this difference was not found at the pulmonary level, in connection with the absence of difference observed on the incidence of severe form and the lethality of the infection compared to the wild type D614 [37].

Therapeutically, the D614G substitution does not impact the immunodominant epitopes of the S protein. Studies on the difference in seroneutralization between wild type D614 and mutated D614G had not shown any significant difference in the neutralizing activity of antibodies induced by vaccines based on the wild-type S protein [37,38]. No difference in the efficacy of seroneutralization by antibodies raised after an infection with a D614 strain was observed either [30,39].

The advantages provided by D614G, the constant maintenance of one of the RBDs of the S protein in the "up" position, and the stabilization of the S1–S2 complex are such that all the new VOC that have appeared since then are descended from clade B.1 and present this substitution [34].

### 4.2. ALPHA Variant (B.1.1.7)

Following the spread of lineage B.1, new lineages emerged in a context of selection pressure related to the extension of vaccination and post-infectious immunization. These lineages have each selected specific sets of mutations, in an asynchronous and geographically isolated manner, which supports the hypothesis of a convergent antigenic evolution, reinforced by the discovery of some of their mutations in independent lineages [26].

The first VOC to be described is the *Alpha* variant [20,25]. It had mutations that gave it a higher immune escape, such as the 69/70 deletion [40]. This deletion leads to a conformational change at the NTD of S1 that reduces the affinity of immunodominant epitopes to neutralizing antibodies [40,41].

The main mutation that characterized this VOC was the N501Y substitution [42]. This is a mutation in the RBD region that causes an interaction between two aromatic rings and formation of an additional hydrogen bond between the RBD and its receptor ACE2. It results in a decrease of the RBD–ACE2 bond dissociation constant [43,44]. N501Y also participates in the stability of the S protein in the open position [43].

Another mutation whose effect has been studied is the P681H substitution [45]. This substitution is directly adjacent to the FCS sequence and is thought to increase the infectivity of SARS-CoV-2 by affecting membrane fusion mechanisms and direct cell-to-cell transmission [46,47], but no difference could have been demonstrated up to now [48]. Another possibility would be the contribution of resistance to interferon β and to the interferon-mediated immune response, but this has not been formally demonstrated to date either [49].

### 4.3. BETA (B.1.351) and GAMMA (P.1) Variants

The *Beta* and *Gamma* variants were described at the same time as the *Alpha* variant [20]. They also had the N501Y substitution but were distinguished by the absence of the 69/70 deletion and the presence of the E484K and K417N/T substitutions [44,50]. They differed from each other in terms of the countries in which they appeared and the geographical areas in which they spread [27,51].

The K417N/T substitutions affect a neutralizing antibody target epitope of the RBD. These substitutions result in the loss of a salt bridge between RBD and ACE2 and increase the dissociation constant of their binding [52]. It is therefore a deleterious mutation in terms of the infectivity of SARS-CoV-2, having led to a negative natural selection of the strains but selected because of the escape from the humoral response provided in a context of developing immunity in the general population, associated with mutations restoring the infectivity potential of the virus such as N501Y or E484K [53]. Indeed, the E484K substitution affects a contact point between RBD and ACE2 and leads to a conformational rearrangement with a tighter binding interface between RBD and ACE2 and the formation of new hydrogen bonds decreasing the dissociation constant [44]. In addition, the E484K substitution impacts one of the important sites of viral recognition by neutralizing antibodies and decreases their affinity, whether from vaccines, convalescent plasmas, or monoclonal antibody treatments [54,55,56].

The *Alpha*, *Beta*, and *Gamma* variants were more competitive due to their immune escape mutations. Each of these VOC were replaced by the *Delta* VOC.

### 4.4. DELTA Variant (B.1.617.2 and AY Lineages)

This variant was distinguished by its mutations which are all different from the mutations found in the previous VOCs, except for D614G, and the main ones are L452R, T478K, E484Q, and P681R [20].

The *Delta* variant was characterized by a higher competitiveness than the *Alpha* variant [57]. It is more transmissible due to a higher viral load and a shorter intergenerational interval.

The L452R substitution affects the RBD region, close to the interface with the ACE2 receptor [58]. It alters the orientation of the β-sheets participating in the RBD motif and causes the creation of a salt bridge between R454 and D467 responsible for a conformational change and a more stable S protein [59,60]. This conformational change decreases interactions with certain neutralizing antibodies and impacts the cellular immune response by altering the 448–456 region of the S protein [61,62]. This region has an epitope recognized by HLA-A24, allowing escape from part of the HLA-dependent immune system (MHC class I) and promoting progression of the infection [63,64].

The T478K substitution results in the replacement of a neutral amino acid by basic amino acid with positive polarity at the RBD–ACE2 interface, increasing the interaction strength between RBD and ACE2 [65]. The association of T478K with L452R also results in conformational change and immune escape to certain neutralizing antibodies as well as increased stability of the S protein [60].

It was observed that the combination of L452R with E484Q increases the strength of RBD–ACE2 binding and thus the infectivity of the virus particle [66].

The P681R substitution facilitates cleavage by TMPRSS2 and activation of the fusion peptide, facilitating membrane fusion mechanisms as well as direct transmission between cells [57,67]. This increased transmissibility is associated with an increase in the virulence of the *Delta* variant, affecting young populations previously spared from symptomatic forms and with an increased risk of severe forms and hospitalization, particularly in non-vaccinated populations.

### 4.5. OMICRON Variant (BA.1)

**Figure 1:**
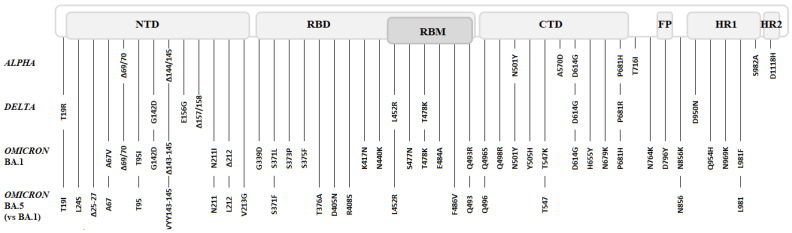
Spike mutations of *Alpha*, *Delta*, *Omicron* BA.1, and *Omicron* BA.5 variants.

The initial *Omicron* variant BA.1, responsible for the fifth wave, was characterized by its large number of mutations: about 50, including 30 in the protein S gene (cf. Figure 1). Some of these mutations were common to those of the *Delta* variant (G142D, T478K, and D614G substitutions) but it also had mutations present in the first three variants of concern (K417N, N501Y, and P681H substitutions, and the 69/70 deletion) as well as other mutations already detected in variants under surveillance such as S477N (*Iota*) or T95I (*Iota/Kappa* variants).

The *Omicron* variant is characterized by higher transmissibility and greater immune escape than the *Delta* variant, responsible for its extremely rapid replacement and the near-exclusive circulation of *Omicron* worldwide since late January 2022.

The RBD of the *Omicron* variant BA.1 had a three-fold higher affinity for ACE2 than the one observed for *Delta* or for the ancestral Wuhan-Hu-1 strain [68]. This difference results from the combined effects of all the mutations present in the S gene and, in particular, the 15 mutations that are located in the RBD region. For example, substitutions Q493R and Q498R create additional electrostatic interactions and S477N an additional hydrogen bond between the RBD and ACE2 [69].

Several mutations in the S gene also participate in its immune escape: certain substitutions at the level of immunodominant epitopes lead to a decrease or even resistance to certain monoclonal antibodies. This is the case of substitutions K417N, E484A, S477N, or Q493R which led to resistance to RONAPREVE (casirivimab/imdevimab), previously effective against the *Delta* variant [69,70], or the N440K substitution which leads to the loss of a strong hydrogen bond with certain amino acids present on some monoclonal antibodies including imdevimab [70]. The substitutions S371L, S373P, and S375F stabilize the RBD of the S protein at the “one-up” position, even after binding to the ACE2 receptor, and reduce the accessibility to certain monoclonal antibodies [71].

The *Omicron* variant is also characterized by the absence of increased morbidity and mortality that accompanied its increased transmissibility. It is predominantly responsible for mild symptomatic forms, with retained vaccine protection against severe forms and altered cellular entry mechanisms that impact viral replication capacity and cell tropism [72]. Indeed, three substitutions are upstream of the FCS (H655Y, N679K, and P681H): some models predict that they would facilitate cleavage at the FCS despite impaired cleavage capacity by TMPRSS2 [68,73]. This alteration favors viral entry by endocytosis and decreases viral penetrance in TMPRSS2-expressing cells, such as those in the lung parenchyma [68]. This modification of the tissue tropism, associated with the lower viral load and the decrease of the direct contamination capacity between cells linked to TMPRSS2, contributes to the decrease of the virulence of the *Omicron* variant despite its higher transmissibility [68].

CTD: C-terminal domain; FP: fusion peptide; HR: heptad repeat; NTD: N-terminal domain; RBD: receptor binding domain; RBM: receptor binding motif.

### 4.6. Omicron’s Sublineages (BA.2 to BA.5)

The *Omicron* variant was the first from which new sublineages emerged independently of the B.1 strain and in turn became the majority. The first circulating *Omicron* variant was reclassified BA.1 (PANGO nomenclature) and consecutive numbers were used to distinguish the new sublineages, retaining the BA prefix [20]. These sublineages are characterized by a few different mutations as well as a return to the wild type in some cases (cf. Figure 1), and by differences in sensitivity to the different treatments available.

The BA.2 lineage became the majority in the world thanks to a higher secondary attack rate of 39% versus 29% for BA.1 and a shorter inter-generational interval. The virulence of the BA.2 sublineage was similar to that of BA.1. Moreover, even if escape from neutralization was observed in vitro [74,75], the efficacy of the vaccine protection against severe forms of COVID-19 was preserved in clinic [76]. On the other hand, from a therapeutic point of view, there was a change in the sensitivity to certain monoclonal antibodies, whether they were effective against BA.1 and had become inactive against BA.2 (such as XEVUDY: sotrovimab) or vice versa (such as EVUSHELD: tixagevimab/cilgavimab) [75]. Cases of reinfection with BA.2 in patients with a history of BA.1 infection had been rare.

Sublineage BA.5 replaced BA.2 in April 2022 due to increased affinity for ACE2 and increased immune escape, probably related to selection of the L452R substitution [75,77,78], one of the major mutations responsible for the increased transmissibility of the *Delta* variant, as well as the 69/70 deletion, the F486V substitution, and reversion to the wild type amino acid at position Q493. Treatment sensitivity and vaccine efficacy remained similar.

Since July 2022 the BA.5 subvariant has remained the majority variant but has evolved into different sublineages. As of November 2022, the major circulating BA.5 sublineages are BF.7 which selected the R346T substitution and BQ.1.1 which selected the additional mutations K444T and N460K [29,79]. In vitro studies showed a decrease in the neutralization of BQ.1.1 compared to BA.5, either by post-infection or post-vaccination antibodies [80,81], although this was better after a booster with a bivalent BA.5 vaccine [82]. This immune escape also affects monoclonal antibodies with a complete loss of efficacy of EVUSHELD that was active against BA.5 [83].

## 5. Conclusions

SARS-CoV-2 is responsible for COVID-19 pandemic that has been ongoing since December 2019. The COVID-19 pandemic has had a colossal global impact. The health systems of each affected country as well as their economies have been challenged, and many regions and even countries economically had to do a halt during the initial waves.

This virus has been studied in great detail, particularly in terms of genomics. In more than two and a half years of pandemic, the major impact of substitutions, the constant evolution of the virus, and the adaptation of its fitness to its host in a context of immune pressure have been highlighted. SARS-CoV-2 has shown a dynamic evolution to the current majority variant: the highly transmissible *Omicron* BA.5* variant.

The circulation of the virus in a population, under pressure of immune selection, raises the issue of the appearance of new variants. Genomic monitoring of SARS-CoV-2 remains essential in order to detect possible genetic changes that could again modify its clinical and epidemiological characteristics, associated with the epidemiological monitoring of severe forms of COVID-19 and the evolution of their sensitivity to available treatments and vaccine efficacy.

## Figures and Tables

**Table 1:** SARS-CoV-2 major Spike amino-acid substitutions and their virological and clinical impacts.

WHO	Mutations of Interest		
Classification	Transmissibility *	Virulence *^,^**	Immune Escape *
	+		+
***Alpha***			
	N501Y		Δ69/70, P681H
	+		+
***Beta***			
	N501Y, E484K, K417N		K417N, E484K
	+		+
***Gamma***			
	N501Y, E484K, K417T		K417T, E484K
	++	+++	++
***Delta***			
	L452R, T478K, P681R	P681R	L452R, T478K, E484Q
	+++	-	+++
			
***Omicron***	S477N, Q493R,		Δ69/70, S371L, S373P,
**BA.1**	Q498R, N501Y		S375F, K417N, N440K,
			S477N, E484A, Q493R
***Omicron*** **BA.5**	+++	-	+++
		
	L452R, Q493		L452R

* +, ++, or +++: low, moderate, or high increase in transmissibility, virulence, and/or immune escape compared to the Wuhan SARS-CoV-2 strain. **: Decrease in virulence compared to the Wuhan SARS-CoV-2 strain.

## Data Availability

Not applicable.
